# Efficacy and Safety of Lecanemab in Treating Alzheimer's Disease: A Real‐World Study in South China

**DOI:** 10.1002/alz70859_101567

**Published:** 2025-12-25

**Authors:** Wang Liao, Qun Yu, Bin Chen, Zhiming Li, Yingte Wang, Ying Yang, Haobo Chen, Haiqun Xie, Xiaoya Gao, Liangyu Zou, Jipeng Ouyang, Shunjun Feng, Feiqi Zhu, Zhou Liu, Hangyu Weng, Lin Han, Jun Liu

**Affiliations:** ^1^ Department of Neurology, the Second Affiliated Hospital of Guangzhou Medical University, Institute of Neuroscience, the Second Affiliated Hospital of Guangzhou Medical University, Guangzhou, Guangdong China; ^2^ Department of Radiology, the Second Affiliated Hospital of Guangzhou Medical University, Guangzhou, Guangdong China; ^3^ Department of Neurology, The First Affiliated Hospital, Jinan University, Guangzhou, Guangdong China; ^4^ Guangzhou First People's Hospital, South China University of Technology, Guangzhou, Guangdong China; ^5^ Foshan Hospital of Sun Yat‐Sen University, Foshan, Guangdong China; ^6^ Department of Neurology, Department of Pediatric Neurology, Zhujiang Hospital of Southern Medical University, Guangzhou, Guangdong China; ^7^ Department of Neurology, Shenzhen People’s Hospital (First Affiliated Hospital of Southern University of Science and Technology), Second Clinical College, Jinan University, Shenzhen, Guangdong China; ^8^ Shunde Hospital, Southern Medical University (The First People’s Hospital of Shunde, Foshan, Foshan, Guangdong China; ^9^ Department of Neurology, Guangdong Provincial People’s Hospital, Guangzhou, Guangdong China; ^10^ Cognitive Impairment Ward of Neurology Department, The Third Affiliated Hospital of Shenzhen University Medical College, Shenzhen, Guangdong China; ^11^ The Affiliated Hospital of Guangdong Medical University, Zhanjiang, Guangdong China; ^12^ Department of Neurology, Dongguan People’s Hospital (Affiliated Dongguan Hospital, South Medical University), Faculty of Neurology, Guangdong Medical University, Dongguan, Guangdong China; ^13^ Department of Neurology, Houjie Hospital of Dongguan, Dongguan, Guangdong China

## Abstract

**Background:**

Lecanemab is the only monoclonal antibody currently available that targets soluble amyloid‐beta (Aβ) protofibrils. It was introduced in China in 2024, but its real‐world efficacy remains to be demonstrated. This study aims to evaluate the efficacy and safety of Lecanemab in real‐world practice in South China.

**Method:**

This was an 18‐month, multicenter, single‐arm, open‐label, prospective observational study conducted across 25 centers. We plan to recruit 500 patients aged 50 to 90 years with mild to moderate AD within 1 year. Participants were treated with Lecanemab (10 mg/kg) biweekly. Evaluations included neuropsychological assessments, plasma biomarkers (Aβ42, Aβ40, GFAP, t‐Tau, p‐Tau217, p‐Tau181, GFAP using Simoa), and other laboratory tests. Amyloid‐Related Imaging Abnormalities (ARIA) were assessed using magnetic resonance imaging (MRI).

**Result:**

A total of 160 participants were recruited (58.75% female, 41.25% male) as of January, 2025, with a mean age of 68.8 ± 9.1 years. Baseline Mini‐Mental State Examination (MMSE) score was 17.3 ± 6.5, Clinical Dementia Rating‐Sum of Boxes (CDR‐SB) score was 5.8 ± 3.6. Regarding APOE ε4 status, 55.63% were carriers, including 18 homozygotes (11.25%). As of now, 21 participants have completed two blood biomarker tests. The baseline levels of blood biomarkers included Aβ42(3.54 ± 2.30 mmol/L), Aβ40( 88.93 ± 39.67 mmol/L), Aβ42/40(0.039 ± 0.014), GFAP(234.84 ± 161.76 mmol/L), and p‐Tau217( 0.99 ± 0.55mmol/L). At 14 weeks, biomarkers showed some improvement: Aβ42(5.71 ± 2.68 mmol/L), Aβ40(144.08 ± 46.86 mmol/L), Aβ42/40(0.037 ± 0.009), GFAP(215.38 ± 144.42mmol/L) and p‐Tau217( 0.92 ± 0.41 mmol/L). The most common adverse events were infusion‐related reactions (22.5%), and ARIA occurred in 15 cases (10.0%), with only 6 cases being symptomatic.

**Conclusion:**

Lecanemab was generally well‐tolerated and the improvements in blood biomarkers suggest that it may have a beneficial impact on disease progression.

